# A New Form of Combined Hyperactive Dysfunction Syndrome: A Unique Case

**DOI:** 10.7759/cureus.80639

**Published:** 2025-03-15

**Authors:** Christian Messina

**Affiliations:** 1 Department "G. F. Ingrassia" Section of Neuroscience, University of Catania, Catania, ITA

**Keywords:** combined hyperactive dysfunction syndrome, glossopharyngeal, neuralgia, occipital, trigeminal

## Abstract

Pain is an unpleasant sensory and emotional experience associated with actual or potential tissue damage. There are different types of pain. Neuropathic pain is caused by a lesion or pathological condition of the somatosensory nervous system. One of the most well-known neuropathic pains is trigeminal neuralgia (TN). However, occipital neuralgia (ON) and glossopharyngeal neuralgia (GPN) exist. These three rare pathologies occur in a very small part of the global population. Combined hyperactive dysfunction syndrome is a rare disorder characterized by overactivity in different cranial nerves, TN, hemifacial spasm, and GPN. In this paper, we present a very rare case of a young woman affected by an association of overactivity in the trigeminal, occipital, and glossopharyngeal nerves, leading to TN, ON, and GPN simultaneously.

## Introduction

According to the International Association for the Study of Pain, pain is an unpleasant sensory and emotional experience associated with actual or potential tissue damage [1]. When the nervous system is damaged peripherally or centrally, sensory loss in the innervation territory of damaged nerves or in those body parts that correspond to a spinal cord or brain occurs [1]. This condition is defined as “neuropathic pain” [1]. Neuralgias are characterized by pain in the cranial or spinal nerve distribution, usually in the cervical roots [2]. Typically, they are characterized by brief, paroxysmal, and painful attacks described as "stabbing" or "ice-pick," although continuous neuropathic pain may occur [2]. The most frequent conditions involve the trigeminal and occipital nerves, whereas less common neuralgias include glossopharyngeal, superior laryngeal, auriculotemporal, and nervus intermedius neuralgia, more often being idiopathic or postherpetic [2]. The diagnosis of neuropathic pain is difficult if the clinician doesn’t recognize the triggering cause, and a lot of diagnostic exams are performed to define the etiology. Combined hyperactive dysfunction syndrome (CHDS) is defined as the combination of hyperactivity symptoms in cranial nerves, specifically trigeminal neuralgia (TN), hemifacial spasm (HFS), and glossopharyngeal neuralgia (GPN), without an explanatory structural lesion [3]. This paper shows the case of an Italian girl suffering from a different and probably new form of CHDS.

## Case presentation

A 29-year-old female patient was diagnosed with TN in 2012, presenting with daily episodes of sensation of “electrical shock” in the left maxillary region of the face, triggered by cold and makeup, which began after a one-month Epstein-Barr virus (EBV) infection and consequent pharyngitis superinfection. After one year, she reported seasonal episodes of “electrical shock” on the left nuchal region, receiving a diagnosis of ON. Subsequently, in 2015, she began to present episodes of pain on the left tonsillar pillar, spreading on the ipsilateral eustachian tube, triggered by voice emission, eating, and swallowing, and was diagnosed with left GPN. In September 2021, she underwent microvascular decompression (MVD) of the left occipital nerves in conflict with the superior cerebellar artery (SCA), reporting no clinical benefit. The neurological examination was within normal limits. Cerebrospinal fluid (CSF) analysis showed no abnormalities (Table 1). Blood tests executed between 2012 and 2015, comprising blood count, liver and renal function, electrolytes, creatine phosphokinase, lactate dehydrogenase, thyroid function, antinuclear antibodies, extractable nuclear antigens screening, serum gangliosides, C-reactive protein, serum glucose, serum amylase and lipase, and infection screening (herpes simplex virus 1 and 2, varicella-zoster virus, cytomegalovirus, EBV, toxoplasma, syphilis, tuberculosis), were unremarkable, except for a mild chronic lymphocytosis and positive serum EBV viral capsid antigen immunoglobulins (Table 2).

**Table 1:** CSF analysis CSF: cerebrospinal fluid, IgG: immunoglobulin G

CSF analysis	Results	Normal values
Proteins	323 mg/L	135-500 mg/L
Glucose	69 mg/dL	<75 mg/dL
Cells	1/µL	<5/µL
IgG	63 mg/dL	-
IgG index	0.52	<0.7
Anti-gangliosides IgM antibodies	Negative	Negative
Anti-gangliosides IgG antibodies	Negative	Negative

**Table 2:** Last performed blood tests with their results Hb: hemoglobin, MCV: mean corpuscular volume, WBC: white blood count, CRP: C-reactive protein, LDH: lactate dehydrogenase, AST: aspartate aminotransferase, AST: alanine aminotransferase, CPK: creatine phosphokinase, TSH: thyroid-stimulating hormone, ANA: antinuclear antibodies, ENA: extractable nuclear antigens, IgM: immunoglobulin M, IgG: immunoglobulin G, HSV1: herpes simplex virus 1, HSV2: herpes simplex virus 2, VZV: varicella-zoster virus, CMV: citomegalovirus, EBV: Epstein-Barr virus, VCA: antiviral capsid antigen, VDRL: venereal disease research laboratory, FTA-ABS: fluorescent treponemal antibody absorption, IGRA: interferon-gamma release assay

Lab tests	Results	Normal values
Hb	12.5 g/dL	10.5-13.5 g/dL
MCV	87 fL	80-100 fL
WBC	5.7 x 10^9^/L	4.0-11.0 x 10^9^/L
Neutrophils	3.7 x 10^9^/L	1.8-7.5 x 10^9^/L
Lymphocytes	4.7 x 10^9^/L	1.0-4.0 x 10^9^/L
CRP	0.6 mg/L	<5 mg/L
LDH	137 U/L	135-225 U/L
Sodium	137 mmol/L	135-145 mmol/L
Potassium	3.7 mmol/L	3.5-5.3 mmol/L
Glucose	75 mg/dL	69-105 mg/dL
Urea	5.2 mmol/L	2.5-7.8 mmol/L
Creatinine	0.82 mg/dL	<1.10 mg/dL
AST	13 U/L	8-33 U/L
AST	27 U/L	15-40 U/L
Lipase	35 U/L	14-72 U/L
Total amylase	75 U/L	30-118 U/L
CPK	89 U/L	29-168 U/L
TSH	4.7 μU/mL	2-10 μU/mL
ANA	1:40	<1:80
ENA screening	Negative	Negative
Anti-gangliosides IgM antibodies	Negative	Negative
Anti-gangliosides IgG antibodies	Negative	Negative
HSV1 IgM	8.9 U/mL	<20 U/mL
HSV1 IgG	23 U/mL	<20 U/mL
HSV2 IgM	6.5 U/mL	<20 U/mL
HSV2 IgG	12.5 U/mL	<20 U/mL
VZV IgM	9.2 U/mL	<25.5 U/mL
VZV IgG	27.2 U/mL	<25.5 U/mL
CMV IgM	0.5 U/mL	<18 U/mL
CMV IgG	2.3 U/mL	<18 U/mL
EBV VCA IgM	22 U/mL	<20 U/mL
EBV VCA IgG	127 U/mL	<35.9 U/mL
Toxoplasma IgM	1.2 IU/mL	<9 IU/mL
Toxoplasma IgG	7.3 IU/mL	<9 IU/mL
VDRL	Negative	<1:16
FTA-ABS	Negative	Negative
IGRA	Negative	Negative

**Figure 1:**
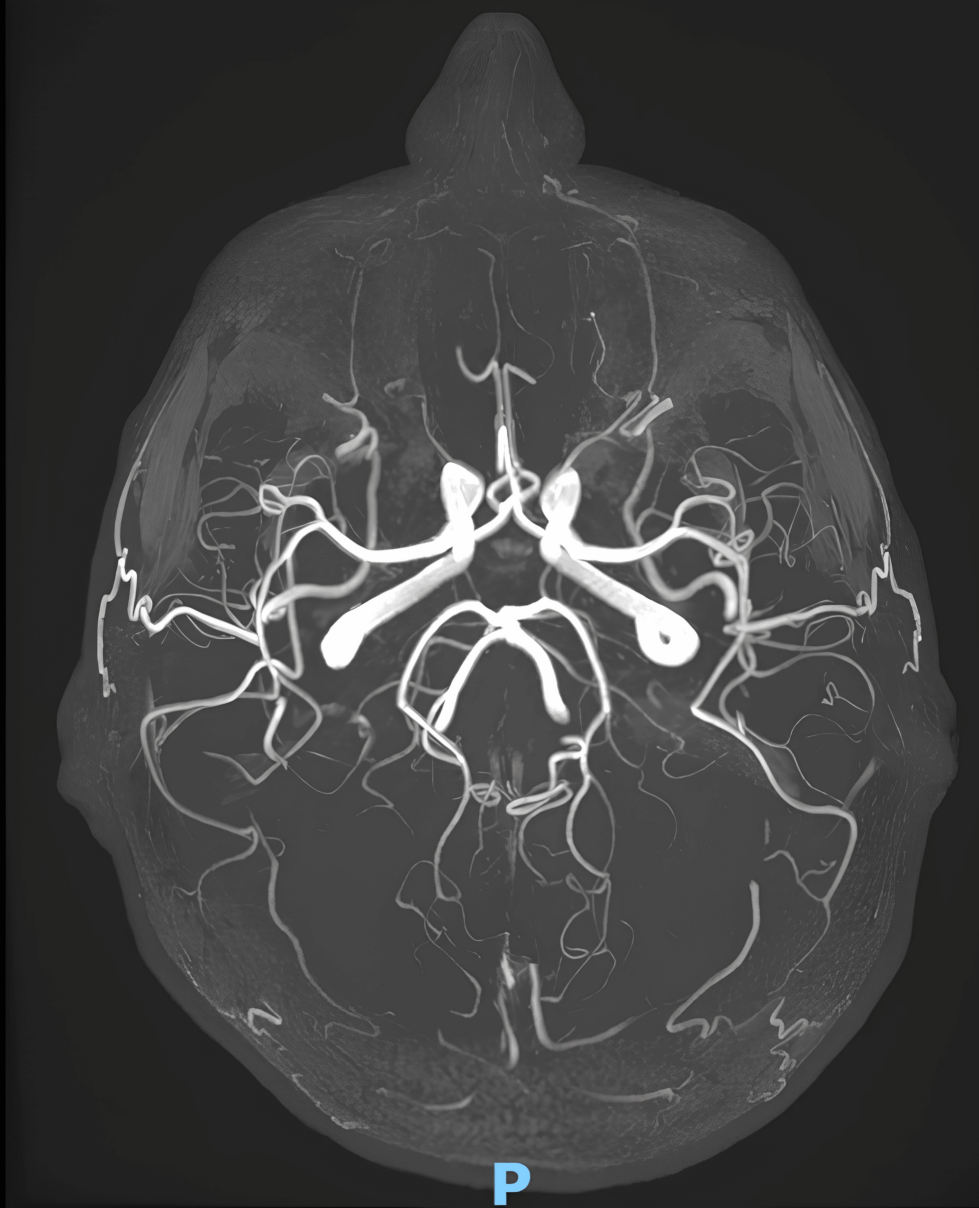
MRA performed in October 2022 MRA: magnetic resonance angiography

Her cardiologic and gastrointestinal evaluations and several brain magnetic resonance imaging exams, with magnetic resonance angiography, were unremarkable (Figure 1). She was treated with carbamazepine, but it gave her only a little benefit in TN.

## Discussion

This is a case of a young woman affected by TN, ON, and GPN occurring simultaneously. TN is characterized by recurrent attacks of neuropathic lancinating facial pain in the dermatomal distribution of the trigeminal nerve [4]. TN is a rare condition with a female-to-male ratio of 3:1, and it is characterized by short-lasting episodes of sharp, lancinating, or electric-like pain occurring in one or more branches of the trigeminal nerve, more often affecting the maxillary or the mandibular branches [4]. Classical TN is caused by neurovascular compression, most frequently by the SCA. However, the compression may be caused even by the anterior inferior cerebellar artery, the basilar artery, and the pontine veins [4]. However, other rare neuropathic conditions are occipital neuralgia and GPN, which affect the posterior region of the head and the sensory distribution of the ninth cranial nerve [5,6]. When only one nerve is affected, we define it as hyperactive dysfunction syndrome (HDS), whereas CHDS is defined as a condition characterized by the involvement of more than one nerve. A few cases of CHDS, which is an extremely rare neurological syndrome characterized by the combination of symptoms arising from overactivity in cranial nerves, and particularly the accompanying mix of TN, HFS, and GPN, without any neurovascular compression evidence [7], have been previously described, occurring mainly in females [7,8]. There are only very few cases of triple vascular compression neuralgia. The prevalence of this disorder was reported as 2.8-2.97% of all patients with HDS [9], with the presenting symptom being HFS or TN; GPN onset has been reported only in one case [9]. MVD has been reported ineffective in CHDS, even if some cases showed clinical benefit after the procedure [7,9,10], as in the presented case. Perez-Roman et al. described the unique case of a 66-year-old male presenting with combined simultaneous unilateral right-sided TN, GN, and hemifacial spasm because of a dolichoectatic vertebrobasilar system compressing the exit zones of the right trigeminal, facial, and glossopharyngeal nerves; this patient underwent microvascular triple decompression, which gave him immediate relief [11]. Narrower posterior fossa, cardiovascular hypertension, and low CSF volume have been reported as comorbidities, above all in females compared to males [9]. However, as in our reported case, the patient did not suffer from hypertension, CSF analysis was unremarkable, and her posterior fossa volume and anatomy were within the range limits.

## Conclusions

Neuralgia is an unpleasant disorder that should be treated to improve the quality of life. Moreover, CHDS is a rarely occurring syndrome usually observed in older females, and people suffering from CHDS complain about a terrible quality of life. This case report highlights the association of the overactivity of the trigeminal, glossopharyngeal, and occipital nerves, which has never been reported. Due to this new association, a new form of CHDS might be described for the first time. Further evaluations about diagnosis and treatment need to be assessed.
